# Fiber degrading enzymes increased monosaccharides release and fermentation in corn distillers dried grains with solubles and wheat middlings steeped without or with protease

**DOI:** 10.1093/tas/txaa153

**Published:** 2020-08-11

**Authors:** Youngji Rho, Rob Patterson, Iris Joye, Mario Martinez, E James Squires, Elijah G Kiarie

**Affiliations:** 1 Department of Animal Biosciences, University of Guelph, Guelph, Ontario, Canada; 2 Canadian Bio-Systems Inc. Calgary, Alberta, Canada; 3 Department of Food Science, University of Guelph, Guelph, Ontario, Canada; 4 School of Engineering, University of Guelph, Guelph, Ontario, Canada

**Keywords:** distillers dried grains with solubles, fermentation, fiber degrading enzymes and protease, fiber solubilization, pretreatment, wheat middlings

## Abstract

Treating fibrous feed ingredients with exogenous feed enzymes may improve their utilization in monogastric animals. An in vitro study was conducted to determine the effects of steeping corn distillers dried grains with solubles (**DDGS**) or wheat middlings (**WM**) with exogenous feed enzymes. Four treatments were arranged as follows: 1) co-product steeped with water (**CON**), 2) CON plus 0.5-g fiber degrading enzymes (**FDE**), 3) CON plus 0.5-g protease (**PRO**), and 4) CON plus 0.5-g FDE and 0.5 g PRO (**FDEPRO**). The FDE contained about 62,000, 37,000, and 8,000 U/g of xylanase, cellulase, and β-glucanase, respectively, whereas activities in PRO amounted to 2,500,000, 1,300,000, and 800,000 U/g of acid, alkaline, and neutral proteases, respectively. Briefly, 50 g of DDGS or WM samples (*n* = 8) were mixed with 500-mL water with or without enzymes and steeped for 0 to 72 h at 37 °C with continuous agitation. The pH, concentration of monosaccharides, and organic acids in the supernatant and apparent disappearance (**AD**) of fiber in solids were measured at 0, 12, 24, 48, and 72 h. There was treatment and time interaction (*P* < 0.005) on monosaccharides concentration. At 12 h, arabinose and glucose concentrations were similar (*P* > 0.05) between FDE and FDEPRO but higher (*P* = 0.002) than for CON in DDGS. For WM, FDE, and FDEPRO had higher (*P* < 0.001) xylose concentration than CON and PRO, whereas glucose concentration was higher (*P* < 0.001) for enzymes than CON at 12 h. However, FDEPRO had higher (*P* < 0.001) xylose concentration than CON, whereas xylose concentration for FDE and PRO was intermediate at 24 h. There was an interaction (*P* < 0.05) between treatment and time effect on lactic acid concentration in DDGS and WM (*P* < 0.005), and acetic acid concentration in WM (*P* < 0.001). In general, monosaccharide concentration was higher between 12 and 24 h and decreased after 48 h, whereas the pH decreased, and concentration of organic acids increased continuously over time (*P* < 0.05). The AD of NDF and ADF in DDGS was greater (*P* = 0.001) for FDE and FDEPRO than CON and PRO at 72 h. In WM, enzymes increased (*P* = 0.007) AD of NDF relative to CON at 72 h. Nonetheless, greater (*P* < 0.05) AD of fiber was observed between 48 and 72 h. In conclusion, although there were differences in responses among co-products, fiber degrading enzymes increased release of fermentable monosaccharides from co-products at 12 to 24 h of steeping and these effects were not extended with the addition of protease.

## INTRODUCTION

Utilization of co-products such as corn distillers dried grains with solubles (**DDGS**) and wheat middlings (**WM**) in monogastric farm animal diets has become more common (Woyengo et al., 2014). Compared with cereal grains, these co-products have higher crude protein and fat concentrations (Casas et al., 2018; Loy and Lundy, 2019). However, they are fiber-rich, a property that negatively influences digestion resulting in decreased feed efficiency (Urriola et al., 2010; Rosenfelder et al., 2013). The major fiber fractions in cereal co-products are arabinoxylans, cellulose, and mixed-linkage β-glucans (de Lange, 2000; Jaworski et al., 2015). Monogastric animals such as pigs and poultry do not secrete endogenous fibrolytic enzymes to degrade fiber and exhibit variable microbial fermentation in the hindgut (Choct et al., 1996; Jorgensen et al., 1997; Leung et al., 2018). The gastrointestinal tract is colonized with diverse microbiota, and the different exogenous and endogenous substrates that are available for fermentation result in diverse metabolites (Brooks et al., 2011; Brestenský et al., 2017). It has been shown that less than 40% of the fiber in cereal co-products is fermentable (Just et al., 1983; Jha and Berrocoso, 2015; Jaworski and Stein, 2017). The high concentration of insoluble relative to soluble fiber is the main challenge in the utilization of fibrous cereal co-products in monogastric animals because insoluble fiber fractions are hydrophobic, crystalline, and recalcitrant to microbial fermentation (Bach Knudsen, 2011; Bach Knudsen, 2014). In addition, cereal fiber has a complex structure as it is formed as heteropolysaccharides with varying substituent groups (Paloheimo et al., 2010). Therefore, the various monosaccharide composition and linkages that are involved determines the functional properties and physiological effects of different dietary fiber sources (Cui et al., 2011).

Soaking these fibrous co-products with exogenous fiber degrading enzymes (**FDE**) allows pre-digestion of the fibrous substrates. Liquid feeding has been reported to improve feed efficiency in pigs compared with dry feeding (Lyberg et al., 2006; Hurst et al., 2008). Further improvement in feed efficiency was achieved by applying FDE to fermented or nonfermented liquid feed compared with the control feed (Rho et al., 2018b). The results of this study suggested that FDE could be a good strategy to solubilize insoluble fiber fractions to enhance fermentability in the animal’s large intestine. For example, steeping undigested ileal contents collected from pigs fed 96% corn or wheat DDGS diets with xylanase (2,000 to 20,000 U/kg) increased the release of soluble arabinoxylans in a dose-dependent manner irrespective of substrate source (Walsh et al., 2016). However, the results of liquid feeding with enzymes vary with different feedstuffs. When pigs were fed liquid steeped DDGS or WM based diet, an improved digestibility of NDF was observed in DDGS steeped with xylanase compared with DDGS steeped without xylanase (Moran et al., 2016). However, there was no effect of xylanase in steeped WM (Moran et al., 2016).

It is imperative to select and match enzymes used during steeping to target-specific polysaccharides, as dietary fiber structure and composition may vary widely (Jaworski et al., 2015; Kiarie et al., 2016a). Moreover, steeping time is also an important factor as time can change the nutrient and microbial profile of the liquid feed. High lactic acid concentrations and low pH is considered an ideal liquid feed (Brooks et al., 2003). Mixing feed or ingredient with water alone is not enough to reach these parameters. Previous studies reported that higher lactic acid concentration in liquid feed resulted from steeping for 10 to 72 h (Brooks et al., 2001; Wiseman, 2016). However, to achieve ideal liquid feed, careful monitoring is required to prevent unwanted pathogen growth as such contaminants may lead to the production of high acetic or ethanol instead of lactic acid (Missotten et al., 2015).

Plant cell walls are mainly composed of cellulose microfibrils that are intertwined with hemicellulose, lignin, and structural proteins (Berglund et al., 2016). In co-products, the proportion of cell wall material is significantly greater than in the parent grains (Stein and Shurson, 2009). Corn and wheat DDGS subjected to in vitro digestion and fermentation simulation without or with a mixture of FDE and protease revealed aggregates of protein–fiber–starch matrix material in both DDGS types as well as different responses to the enzyme application (Jha et al., 2015). Microscopy visualization revealed that DDGS was more porous than WM, making enzymes more effective (Jha et al., 2015). These results suggested that applying a combination of FDE and protease may be more effective in breaking down complex structures. This research is in its infancy and additional screening of FDE and protease activities are required to develop specific enzyme products to be supplemented to the feed to increase fiber solubilization.

The objective of the present study was to determine the effect of applying FDE containing xylanase, β-glucanase, and cellulase activities with or without protease in steeped DDGS or WM on the release of monosaccharides, fermentation, and apparent disappearance (**AD**) of fiber fractions. It was hypothesized that fiber hydrolysis will be increased by applying a combination of FDE and protease relative to what is observed for FDE or protease alone.

## MATERIALS AND METHODS

### Feed Ingredient Samples and Treatments

The sample of DDGS used was from IGPC Ethanol Inc. (Aylmer, Ontario, Canada) and corn, wheat, and WM samples from Floradale Feed Mill Limited (Floradale, Ontario, Canada). The samples were used as received, without further processing. The mean particle size and SD of DDGS and WM were 271.80 ± 6.58 and 203.05 ± 9.86 µm, respectively. Each co-product (DDGS and WM) was tested in an independent experiment. Four treatments were tested per co-product: 1) co-product steeped with water excluding enzymes (**CON**), 2) CON with 0.5-g FDE (**FDE**) 3) CON with 0.5-g protease (**PRO**), and 4) CON with 0.5-g FDE and 0.5 g protease (**FDEPRO**). This was in alignment with the manufacturer's recommendation on the substrate to enzyme ratio. Enzyme activities in FDE were 62,000 U/g of xylanase, 8,000 U/g of β-glucanase, and 37,000 U/g of cellulase. The enzyme activity for PRO was 2,500,000 U/g of acid protease, 800,000 U/g of neutral protease, and 1,300,000 U/g of alkaline protease. The enzyme mixtures and enzyme activity data were provided by Canadian Bio-systems Inc. (Calgary, Alberta, Canada).

### Experimental Procedures

In sterile plastic bottles, 50 g of DDGS or WM was weighed and hand-mixed with 500 mL of deionized water with or without enzymes. The bottles were sealed tightly with their screw cap to limit additional air coming into the bottle. The samples were incubated in a semi-aerobic condition as no additional gas was flushed into the headspace of bottles. Samples were aliquoted to individual bottles for timepoints: 0, 12, 24, 48, and 72 h. A total of four batches of steeping were conducted with two bottles per treatment per batch resulting into eight bottles per treatment at each timepoint. At timepoint 0 h, pH was measured immediately after hand mixing using Fisher Scientific Accumet AB 150 pH meter (Fisher Scientific, Toronto, Ontario, Canada) standardized with certified pH 4.0, 7.0, and 10.0 buffer solution (Fisher Scientific) and the bottles stored at 4 °C for the solids to settle for 12 h. The mixtures for timepoints 12 up to 72 h were incubated at 37 °C with continuous agitation (200 rpm) using a floor incubator (Controlled Environment Incubator Shaker, New Brunswick Scientific, Enfield, CT). At their assigned timepoints, bottles were withdrawn from the incubator, hand-mixed, pH read, and stored at 4 °C for the solids to settle for 12 h. After the solids were settled, the supernatant was carefully transferred into a 15-mL centrifuge tube and centrifuged at 30,000 g for 15 min at 4 °C. Aliquots of the supernatant were transferred into 2-mL tubes and were stored at −20 °C for organic acid and monosaccharides analyses. After the supernatant was sampled the remaining solids and supernatant in the bottle were mixed and transferred into an aluminum container and were freeze-dried. After freeze-drying, the samples were weighed to calculate the AD of dry matter (**DM**) and fiber components (neutral detergent fiber, **NDF** and acid detergent fiber, **ADF**) poststeeping.

### Sample Processing and Laboratory Analyses

The DDGS and WM samples were analyzed for particle size using Ro-Tap Sieve® Shaker (W.S. TylerRX-30E model, Hoskin Scientific Ltd, Burlington, Ontario, Canada). Freeze-dried samples of steeping, DDGS, WM, and independent corn and wheat grain samples were finely ground (<1 mm) with a coffee grinder (KitchenAid, Benton Harbor, MI). Corn grain, DDGS, wheat grain, and WM were analyzed for DM, ash, crude fat, crude protein (**CP**), NDF ADF, sugar monomers, and glycosyl linkages. The freeze-dried steeped co-products were analyzed for DM, NDF, and ADF. The supernatant of steeped DDGS and WM was analyzed for the concentration of organic acids (lactic and acetic) and monosaccharides (arabinose, xylose, and glucose). Dry matter was determined using method 930.15 (AOAC, 2004) and ash content was determined according to method 942.05 (AOAC, 2004). Crude fat content was analyzed using an ANKOM XT 20 Extractor (Ankom Technology, Macedon, NY). Nitrogen was determined with a CNS-2000 carbon, N, and sulfur analyzer (Leco Corporation, St. Joseph, MI) according to the combustion method 968.06 (AOAC, 2004). Crude protein values were calculated by multiplying the analyzed N values by 6.25 (AOAC, 2004). Neutral detergent fiber and ADF concentrations were measured by using the Ankom 200 Fiber Analyzer (Ankom Technology, Macedon, NY) according to Van Soest et al. (1991).

Monosaccharides and glycosyl linkage characterization were carried out using the method described by Pettolino et al. (2012). Monosaccharides were analyzed with gas chromatography equipped with a capillary column SP-2330 (SUPELCO, Bellefonte, PA). Briefly, prior to sample analysis, hydrolysis was conducted with 2.50 M trifluoroacetic acid for 90 min followed by a reduction in a 1.00 M sodium borodeuteride mixed with 2.00 M ammonium hydroxide and an acetylation reaction. The gas chromatography–mass spectrometry (**GC-MS**) conditions were as follows: injector volume, 2.0 μL; injector temperature, 240 °C; detector temperature, 300 °C; carrier gas (helium), velocity 1.9 m/s; split ratio, 1:2; temperature program over the elution profile was 160 °C for 6 min, then 4 °C/min to 220 °C for 4 min, then 3 °C/min to 240 °C for 5 min, and then 11 °C/min to 255 °C for 5 min. The glycosyl-linkage characterization was performed by GC–flame ionization detector–MS (7890A and 5975C inert mass selective detector with a Triple-Axis detector, Agilent Technologies, Inc., Santa Clara, CA) on an SP-2330 capillary column SP-2330 (SUPELCO, Bellefonte, PA). Samples were partially methylated and subsequently hydrolyzed with 2.00 M trifluoroacetic acid. Partially methylated residues were analyzed as partially methylated alditol acetates. The GC-MS was set as follows for analysis: injector volume, 1 μl; injector temperature, 240 °C; detector temperature, 300 °C; carrier gas (helium), velocity of 1.9 m/s; split ratio, 100:1; temperature program was 100 °C for 2 min, and then 8 °C/min to 240 °C for 20 min.

Concentrations of organic acids (lactic and acetic acid) and monosaccharides (arabinose, xylose, and glucose) in supernatants of steeped co-products were analyzed using high-performance liquid chromatography (**HPLC**; Agilent 1100 Series, Agilent Technologies; Leung et al., 2018). Briefly, samples were thawed to room temperature, vigorously mixed by vortexing, and subsequently centrifuged at 30,000 g for 15 min. An aliquot of 160 µL was mixed with 640 µL of 0.005 N H_2_SO_4_ and then filtered with a 13-mm syringe filter (Fisher Scientific) and transferred to HPLC vials. Prepared samples were separated over a 300 × 7.8-mm 8µ Rezex™ ROA-Organic Acid H+ (8%) column (Phenomenex, Torrance, CA). The HPLC was set as follows for analysis: column temperature, 60 °C; injection volume, 20 μl; refractive index detector (Agilent 1100 Series, Agilent Technologies) temperature, 400 °C; mobile phase (0.005 N sulfuric acid) velocity, 0.5 mL/min; cycle time 45 min. The retention times for lactic and acetic acid, glucose, xylose, and arabinose were found to be 16.9, 19.9, 12.5, 13.4, and 14.4 min, respectively.

### Calculations and Statistical Analyses

The AD of DM, NDF, and ADF was calculated using the following equation:

$$\begin{array}{ll} & AD\;(\;\%)=\\ & \left[\frac{Concentration\;before\;steeping-Concentration\;after\;steeping\;\;}{Concentration\;\;before\;\;steeping}\right]\\ & \times \;100 \end{array}$$

All data were analyzed using the GLIMMIX procedure of SAS 9.2 (SAS Inst. Inc., Cary, NC). The model had treatment, sampling time, and their interactions as fixed effect and batches of steeping as a random effect. LSMeans were separated using the Tukey’s test. Data for DDGS and WM were analyzed as independent experiments. Significant differences were declared when *P* < 0.05.

## RESULTS

### Chemical Composition of Corn, Wheat, and Co-products

The chemical composition of the co-products and cereal grain samples are shown in Table 1. The DDGS and WM showed greater ADF, NDF, fat, and CP concentration than the grains. The NDF level was 2.42 times higher for DDGS than for corn, and 2.23 times greater in WM than in wheat. Co-products had ~2.76 times greater ADF concentration than grains. Crude fat and CP contents were 2.68 and 3.77 times higher in DDGS than corn, and 2.79 and 1.48 times higher in WM than in wheat. The A/X was higher for DDGS and WM than corn and wheat. The most abundant glycosyl linkage in corn, wheat, DDGS, and WM was 4-glucose (**Glc**; Table 1). The 4-Glc linkage content of DDGS was ~6.7 times less than in corn grain, while in WM, about 1.6 times less 4-Glc linkages were found than wheat grain. The 4-xylose (**Xyl**) linkage content of corn, wheat, DDGS, and WM was 1.2, 1.2, 0.5, and 2.2%, respectively, while 3,4-Xyl linkages were only detected in DDGS at a level of 2.8%. The content of terminal (**T**)-Ara-f (arabinofuranosyl) groups in corn, wheat, DDGS, and WM was 0.6, 0.2, 2.3, and 0.6%, respectively. The glycosyl linkage of T-galactose (**Gal**) was 0.7, 0.5, 1.2, and 0.8 % in corn, wheat, DDGS, and WM, respectively.

**Table 1.** Chemical and glycosyl linkage composition in corn, wheat, corn distiller’s DDGS and WM, in DM basis

			Co-products	
Item	Corn*	Wheat*	DDGS	WM
DM, %	88.5	88.5	91.3	86.6
Ash, %	1.4	2.0	5.2	4.7
ADF, %	4.6	4.9	12.4	13.7
NDF, %	15.5	17.7	36.5	40.5
Fat, %	3.4	1.6	8.7	4.61
CP, %	8.5	12.5	31.1	18.9
Monosaccharides, %				
Xylose	1.36	1.36	3.61	2.54
Arabinose	1.36	1.13	4.82	2.60
Mannose	0.34	0.34	0.77	0.34
Galactose	0.79	0.56	1.31	0.90
Glucose	35.0	31.2	6.35	26.8
A/X ratio^†^	1.00	0.83	1.33	1.02
Glycosyl linkage, %				
T-Glc	2.71	1.69	1.31	2.37
4-Glc	26.6	23.1	3.83	16.2
3,4-Glc	1.47	0.90	0.44	0.79
4,6-Glc	1.69	1.81	0.77	1.47
3,4,6-Glc	0.23	0.23	0.00	0.11
T-Ara-f	0.68	0.23	2.52	0.68
5-Ara-f	0.68	0.90	2.30	1.36
4-Xyl	1.36	1.36	0.55	2.49
3,4-Xyl	0.00	0.00	3.07	0.00
T-Gal	0.79	0.56	1.31	0.90

^*^Corn and wheat were not parent grains of DDGS and WM.

^†^Arabinose to xylose ratio.

T; terminal, Glc; Glucose, Ara-f; Arabinofuranose, Xyl; xylose, Gal; galactose.

### Monosaccharides Concentration in Supernatant of Steeped Co-products

The monosaccharides concentration in the supernatant of steeped DDGS over time is shown in Table 2. Treatment and time interaction effect were seen in arabinose concentration (*P* = 0.002). At 12 h, the concentration of arabinose was higher for the FDE and FDEPRO relative to CON; however, PRO was not different from FDE, FDEPRO, and CON. Arabinose concentration in PRO was lower than FDE and FDEPRO at 24 h (*P* = 0.002). At 48 and 72 h, the arabinose levels in DDGS were not different among the different treatments. The interaction effect was not seen (*P* > 0.05) for xylose concentration; however, the main effects of treatment and time were observed (*P* ≤ 0.001). The xylose concentration was higher for FDE and FDEPRO than CON and PRO (*P* < 0.01). The highest xylose concentration was observed at 12 h (*P* = 0.001). There was treatment and time interaction in glucose concentration (*P* = 0.002). At 12 and 24 h, FDE and FDEPRO had a higher glucose concentration compared with PRO (*P* = 0.002). However, glucose concentration was not different among treatments at 48 and 72 h (*P* > 0.05). The highest concentrations of arabinose, xylose, and glucose were observed at 12 and 24 h after which concentration generally decreased at 48 and 72 h (*P* < 0.001)

**Table 2.** Effects of FDE without or with protease on concentration of monosaccharides, organic acids (µmol/mL), and pH in the supernatant fractions of steeped corn distillers DDGS over time

Item (µmol/mL)		Arabinose	Xylose	Glucose	Lactic acid	Acetic acid	pH
Treatment^1^*Time^2^							
CON	0	2.15^cde^	3.21	6.66^cd^	2.95^h^	1.53	4.62^ab^
CON	12	1.86^e^	3.41	5.36^cd^	4.19^h^	2.37	4.58^ab^
CON	24	3.30^abcde^	4.41	12.96^bcd^	8.36^h^	2.85	4.48^abc^
CON	48	1.67^e^	1.83	2.29^d^	37.76^defg^	9.77	4.02^ef^
CON	72	2.11^de^	2.47	3.70^d^	50.94^bcde^	13.52	3.94^f^
FDE	0	2.88^bcde^	4.03	21.34^abcd^	3.82^h^	1.66	4.64^a^
FDE	12	5.88^ab^	7.80	37.19^a^	4.39^h^	2.84	4.56^ab^
FDE	24	5.25^abcd^	5.30	24.57^abc^	22.85^fgh^	3.71	4.24^de^
FDE	48	2.14^de^	4.97	8.55^cd^	58.94^abcd^	16.42	3.60^h^
FDE	72	1.73^e^	5.09	7.23^cd^	83.63^a^	17.97	3.52^h^
PRO	0	2.00^de^	3.32	5.66^cd^	3.24^h^	1.22	4.63^a^
PRO	12	2.56^bcde^	4.03	6.34^cd^	5.12^h^	2.45	4.58^ab^
PRO	24	1.66^e^	1.81	2.39^d^	15.87^gh^	4.86	4.40^bcd^
PRO	48	2.05^de^	2.39	2.87^d^	42.07^cdefg^	16.03	4.01^ef^
PRO	72	1.80^e^	1.88	3.34^d^	47.24^cdef^	12.61	3.88^fg^
FDEPRO	0	2.58^bcde^	3.73	19.50^abcd^	3.51^h^	1.82	4.65^a^
FDEPRO	12	5.66^abc^	7.22	31.16^ab^	5.40^h^	3.25	4.61^ab^
FDEPRO	24	6.59^a^	6.25	29.56^ab^	30.55^efgh^	4.08	4.31^cd^
FDEPRO	48	2.56^bcde^	5.26	8.32^cd^	68.26^abc^	17.72	3.69^gh^
FDEPRO	72	1.84^e^	4.99	6.35^cd^	78.60^ab^	20.89	3.60^h^
SEM		1.32	1.79	5.95	8.59	2.78	0.05
Treatment							
CON		2.19	3.03^b^	3.60	20.84	6.01^b^	4.33
FDE		3.57	5.44^a^	19.72	34.72	8.52^ab^	4.11
PRO		2.01	2.69^b^	3.75	22.71	7.43^ab^	4.30
FDEPRO		3.85	5.49^a^	18.92	37.26	9.55^a^	4.17
SEM		1.15	1.57	4.20	7.09	2.28	0.02
Time, h							
0		2.40	3.57^b^	13.29	3.38^d^	1.56^b^	4.63^a^
12		3.99	5.61^a^	20.01	4.78^d^	2.73^b^	4.58^a^
24		4.20	4.44^ab^	17.37	19.41^c^	3.88^b^	4.36^b^
48		2.11	3.61^b^	5.50	51.76^b^	14.98^a^	3.83^c^
72		1.88	3.61^b^	5.15	65.10^a^	16.25^a^	3.73^d^
SEM		1.18	1.60	5.02	7.19	2.35	0.03
*P-*value							
Treatment		<0.001	<0.01	<0.001	<0.001	0.0137	<0.001
Time		<0.001	0.001	<0.001	<0.01	<0.001	<0.001
Treatment*Time		0.002	0.273	0.002	0.005	0.283	<0.001

^1^CON; no enzyme, PRO; CON + protease, FDE; CON + FDE, and FDEPRO: CON + FDE and protease.

Enzyme activities were: FDE: xylanase: 62,000 U/g, β-glucanase: 8,000 U/g, cellulase: 37,000 U/g, proteases: acid 2,500,000 U/g, neutral 800,000 U/g, and alkaline 1,300,000 U/g.

About 50 g of DDGS or WM was mixed with 500 mL of deionized water and 0.5 g of respective enzyme and incubated at 37 °C with agitation.

^2^0; steeped for 0 h followed by 12 h of settling at 4 °C,12; steeped for 12 h followed by 12 h of settling at 4 °C, 24; steeped for h followed by 12 h of settling at 4 °C, 48; steeped for 48 h followed by 12 h of settling at 4 °C, 72; steeped for 0 h followed by 72 h of settling at 4 °C. At each timepoint, LS means with different letters differs (*P* < 0.05), *n* = 8.

Arabinose, xylose, and glucose concentrations at different timepoints of steeping WM are shown in Table 3. Treatment and time interaction effect was observed for all sugars (*P* ≤ 0.005).

**Table 3.** Effects of FDE without or with protease on the concentration of monosaccharides, lactic and acetic acid (µmol/mL), and pH in the supernatant fractions of steeped WM over time

Item (µmol/mL)		Arabinose	Xylose	Glucose	Lactic acid	Acetic acid	pH
Treatment^1^*Time^2^							
CON	0	6.55^c^	20.47^fghi^	16.14^de^	9.82^j^	4.36^f^	5.81
CON	12	11.52^abc^	36.54^ef^	5.54^ef^	51.67^hi^	28.37^e^	5.06
CON	24	10.74^abc^	8.24^hi^	1.67^f^	118.24^g^	31.47^de^	3.99
CON	48	3.10^c^	5.45^i^	2.01^f^	146.51^efg^	37.05^de^	3.99
CON	72	2.93^c^	5.50^i^	2.21^f^	155.51^def^	46.78^cde^	3.99
FDE	0	3.89^c^	28.17^fg^	26.17^cd^	9.61^j^	3.67^f^	5.85
FDE	12	16.91^ab^	83.49^ab^	38.73^b^	58.50^h^	49.89^cd^	5.09
FDE	24	19.50^a^	63.47^cd^	25.88^cd^	126.69^fg^	77.97^a^	3.95
FDE	48	9.74b^c^	52.84^de^	9.10^ef^	177.84^cde^	83.59^a^	3.87
FDE	72	11.09^abc^	50.89^de^	2.14^f^	222.53^ab^	82.27^a^	3.85
PRO	0	3.90^c^	21.68^fghi^	24.53^cd^	10.46^j^	4.19^f^	5.64
PRO	12	11.30^abc^	50.96^de^	41.26^b^	35.7^hij^	35.71^de^	4.56
PRO	24	5.79^c^	25.24^fgh^	2.87^f^	179.81^cde^	57.97^bc^	3.83
PRO	48	2.76^c^	18.69^fghi^	2.96^f^	192.9^bc^	78.95^a^	3.67
PRO	72	4.43^c^	16.98^ghi^	3.35^f^	190.22^bcd^	94.62^a^	3.64
FDEPRO	0	4.13^c^	28.57^fg^	31.19^bc^	9.53^j^	4.12^f^	5.70
FDEPRO	12	19.22^a^	98.76^a^	56.85^a^	26.48^ij^	33.60^de^	4.74
FDEPRO	24	11.74^abc^	72.77^bc^	6.52^ef^	168.46^cde^	47.53^cde^	3.99
FDEPRO	48	9.30^bc^	67.89^bcd^	2.28^f^	214.33^ab^	74.52^ab^	3.88
FDEPRO	72	8.47^bc^	67.28^bcd^	3.14^f^	242.36^a^	74.50^ab^	3.95
SEM		2	5.03	2.77	7.93	4.81	0.15
Trt							
CON		6.97	15.24	5.51	96.35	29.61	4.45^a^
FDE		12.23	55.77	20.40	119.03	59.48	4.27^b^
PRO		5.64	26.71	14.99	121.84	54.29	4.56^a^
FDEPRO		10.57	67.05	20.00	132.23	46.85	4.52^a^
SEM		0.89	3.42	1.72	4.74	2.82	0.13
Time, h							
0		4.62	24.72	24.51	9.85	4.08	5.75^a^
12		14.74	67.44	35.59	43.11	36.89	4.86^b^
24		11.94	42.43	9.24	148.3	53.74	3.94^c^
48		6.22	36.22	4.09	182.9	68.53	3.85^c^
72		6.73	35.16	2.71	202.66	74.54	3.85^c^
SEM		1.05	3.63	1.86	5.17	3.09	0.13
*P*							
Treatment		<0.001	0.001	<0.001	<0.001	<0.001	<0.001
Time		<0.001	0.001	<0.001	<0.001	<0.001	<0.001
Treatment*Time		0.005	<0.001	<0.001	<0.001	<0.001	0.438

^1^CON; no enzyme, PRO; CON + protease, FDE; CON + FDE, and FDEPRO: CON + FDE and protease.

Enzyme activities were: FDE: xylanase: 62,000 U/g, β-glucanase: 8,000 U/g, cellulase: 37,000 U/g, proteases: acid 2,500,000 U/g, neutral 800,000 U/g, and alkaline 1,300,000 U/g.

About 50 g of DDGS or WM was mixed with 500 mL of deionized water and 0.5 g of respective enzyme and incubated at 37 °C with agitation.

^2^0; steeped for 0 h followed by 12 h of settling at 4 °C,12; steeped for 12 h followed by 12 h of settling at 4 °C, 24;steeped for h followed by 12 h of settling at 4 °C, 48; steeped for 48 h followed by 12 h of settling at 4 °C, 72; steeped for 0 h followed by 72 h of settling at 4 °C. At each timepoint, LSmeans with different letters differs (*P* < 0.05), *n* = 8.

Arabinose concentration increased at 12 and 24 h, and xylose concentration increased at 12 h and subsequently decreased for all treatments (*P* < 0.001). The highest glucose concentration was seen at 12 h for FDE, PRO, and FDEPRO, but for the CON, the highest glucose concentration was observed at 0 h (*P* < 0.001). Treatments with FDE had a higher arabinose concentration in WM at 24 h relative to the PRO; however, CON was not different to FDE, FDEPRO and PRO (*P* = 0.005). Xylose concentrations at 12, 24, 48, and 72 h were greater in FDE and FDEPRO than in the CON and PRO (*P* < 0.001), whereas FDE, FDEPRO, and CON, PRO was not different. The glucose concentration of FDEPRO in steeped WM at 0 h was higher than in CON samples. However, FDE and PRO were not different from CON or FDEPRO at 0 h (*P* > 0.05). At 12 h, the FDEPRO resulted in a higher glucose concentration relative to the other treatments. At 24 h, FDE resulted in a higher glucose concentration relative to the other treatments (*P* < 0.001). Glucose concentrations were similar between treatments from 48 h (*P* > 0.05).

### Lactic and Acetic Acids Concentration and pH in Supernatant of Steeped Co-products

Lactic and acetic acid concentrations and pH over time for steeped DDGS are shown in Table 2. Lactic and acetic acids concentration increased while pH decreased for all treatments over time (*P* < 0.001). There was a treatment and time interaction effect for lactic acid concentration in steeped DDGS (*P* = 0.005). At 0, 12, and 24 h, lactic acid concentration was not different among treatments (*P* > 0.05). At 48 h, FDEPRO had higher lactic acid concentration than CON; however, at 72 h, FDE had a higher lactic acid concentration than CON and PRO (*P* < 0.001). There was no treatment and time interaction on acetic acid concentration, although the main effects of treatment and time were observed (*P* < 0.05). Acetic acid concentration increased over time (*P* < 0.001). Treatment FDEPRO had a higher acetic acid concentration than CON; however, FDE and PRO were not different from FDEPRO or CON (*P* = 0.014). Interaction between treatment and time was seen for the pH of steeped DDGS (*P* < 0.001). There were no pH differences among treatments at 0 and 12 h. At 24-h, CON had a higher pH compared with FDE; however, PRO and FDEPRO were not different from CON and FDE. The pH at 48 and 72 h was lower for FDE and FDEPRO than CON and PRO (*P* < 0.001).

Lactic and acetic acid concentrations and pH for steeped WM are presented in Table 3. There were treatment and time interactions for lactic and acetic acid concentrations (*P* < 0.001). In this context, greater lactic acid concentration was observed for FDE vs. FDEPRO at 12 h, PRO and FDEPRO *vs.* CON and FDE at 24 h and FDEPRO vs. FDE at 48 h. Lactic acid concentration was higher for FDEPRO and FDE than CON; however, PRO was not different from FDE and CON at 72 h of steeping WM (*P* < 0.001). Acetic acid concentration of steeped WM at 0 h was not (*P* > 0.05) different among treatments; however, at 12 h, FDEPRO had greater acetic acid concentration compared with CON, whereas PRO and FDEPRO was not different to FDEPRO and CON (*P* < 0.001). Treatment FDE had the highest acetic acid concentration among treatment at 24 h; however, at 48 and 72 h acetic acid concentration of FDE, PRO, and FDEPRO were not different but higher than CON (*P* < 0.001). Treatment and time interactions were not observed in pH of steeped WM (*P* = 0.438); however, treatment and time effects were independent (*P* < 0.001). The pH was highest at 0 h and decreased over time resulting in the lowest pH at 48 and 72 h (*P* < 0.001). Treatment FDE had the lowest pH compare with others while CON, PRO, and FDEPRO was not different (*P* < 0.001).

### AD of DM, NDF, and ADF in the Residue of Steeped Co-products

Treatment and time interactions were observed for the AD of DM, NDF, and ADF of the residue of steeped DDGS (Table 4, *P* < 0.05). The AD of DM increased over time, showing the greatest AD of DM at 48 and 72 h (*P* < 0.001). There were no differences in AD of DM among the treatments at 0 and 12 h; however, FDEPRO had a greater AD of DM than FDE at 24 h, whereas CON and PRO were not different from FDE and FDEPRO (*P* < 0.001). There were no differences in AD of DM at 48 and 72 h of steeping DDGS (*P* < 0.001). The AD of NDF and ADF of steeped DDGS over time is shown in Table 4. At 0 h, no differences were seen among treatment. The AD of NDF at 12 h was greater in FDE and FDEPRO compared with CON and PRO, while CON and PRO was not different (*P* = 0.001). At 24 h, FDEPRO had a greater AD of NDF than CON, but FDE and PRO was not different to FDEPRO nor CON (*P* = 0.001). The AD of NDF at 48 and 72 h was higher for FDE and FDEPRO than PRO and CON (*P* = 0.001). The AD of ADF of DDGS was not different at 0 h among treatments; however, at 12 h FDEPRO was higher than PRO, whereas CON and FDE were not different from FDEPRO and PRO (*P* = 0.0012). At 24, 48, and 72 h, AD of ADF for FDE and FDEPRO was similar but greater than for CON and PRO (*P* < 0.05), whereas CON and PRO were not different (*P* = 0.0012).

**Table 4.** Effects of FDE with or without protease on AD of DM, NDF, and ADF at different timepoints of steeped distillers DDGS

Item (%)		DM	NDF	ADF
Treatment^1^*Time^2^				
CON	0	8.08^def^	1.25^d^	6.85^d^
CON	12	7.70^ef^	1.25^d^	8.11^cd^
CON	24	8.66^cdef^	1.00^d^	5.65^d^
CON	48	8.77^bcdef^	1.29^d^	5.22^d^
CON	72	9.49^abcde^	3.43^cd^	9.80^cd^
FDE	0	7.26^ef^	3.87^cd^	11.53^cd^
FDE	12	7.43^ef^	7.27^bcd^	12.38^bcd^
FDE	24	7.64^ef^	11.11^abcd^	20.32^abc^
FDE	48	10.23^abcd^	19.35^a^	30.64^a^
FDE	72	11.04^ab^	20.36^a^	27.60^a^
PRO	0	6.60^f^	4.22^cd^	5.80^d^
PRO	12	7.92^def^	4.33^cd^	5.23^d^
PRO	24	8.44^cdef^	5.86^bcd^	6.31^d^
PRO	48	9.27^abcde^	3.86^cd^	6.00^d^
PRO	72	9.26^abcde^	5.40^cd^	5.02^d^
FDEPRO	0	6.72^f^	6.50^bcd^	12.41^bcd^
FDEPRO	12	9.52^abcde^	12.27^abc^	21.23^abc^
FDEPRO	24	10.14^abcd^	16.16^ab^	25.06^ab^
FDEPRO	48	10.63^abc^	18.05^a^	31.19^a^
FDEPRO	72	11.58^a^	19.39^a^	28.57^a^
SEM		0.60	2.62	2.72
Treatment				
CON		8.54	1.64	7.13
FDE		8.72	12.39	20.50
PRO		8.3	4.74	5.67
FDEPRO		9.72	14.48	23.69
SEM		0.44	1.84	1.47
Time, h				
0		7.17	3.96	9.15
12		8.14	6.28	11.74
24		8.72	8.53	14.34
48		9.73	10.64	18.26
72		10.34	12.15	17.75
SEM		0.45	1.90	1.57
*P*-value				
Treatment		<.0001	<.0001	<.0001
Time		<.0001	<.0001	<.0001
Treatment*Time		0.001	0.0012	0.0001

^1^CON; no enzyme, PRO; CON + protease, FDE; CON + FDE, and FDEPRO: CON + FDE and protease.

Enzyme activities were: FDE: xylanase: 62,000 U/g, β-glucanase: 8,000 U/g, cellulase: 37,000 U/g, proteases: acid 2,500,000 U/g, neutral 800,000 U/g, and alkaline 1,300,000 U/g.

About 50 g of DDGS or WM was mixed with 500 mL of deionized water and 0.5 g of respective enzyme and incubated at 37 °C with agitation.

^2^0; steeped for 0 h followed by 12 h of settling at 4 °C,12; steeped for 12 h followed by 12 h of settling at 4 °C, 24;steeped for h followed by 12 h of settling at 4 °C, 48; steeped for 48 h followed by 12 h of settling at 4 °C, 72; steeped for 0 h followed by 72 h of settling at 4 °C. At each timepoint, LSmeans with different letters differs (*P* < 0.05), *n* = 8.

The AD of DM, NDF, and ADF of steeped WM is shown in Table 5. Treatment and time interactions were seen in AD of DM of steeped WM (*P* < 0.001). There was no difference in AD of DM among treatment at 0 and 12 h. However, at 24, 48, and 72 h, FDEPRO, PRO, and FDE had a greater AD of DM than CON (*P* < 0.001). An interactive effect was seen for AD of NDF (*P* = 0.007) such that FDE and FDEPRO had higher AD of NDF than PRO and CON; however, PRO was greater than CON over all timepoints (*P* = 0.007). There was a treatment and time interaction for AD of ADF of steeped WM (*P* < 0.0001). The AD of ADF at 0 and 12 h was not different among treatments; however, FDE and FDEPRO had a greater AD of ADF compared with PRO and CON at 24 h. The AD of ADF at 48 h was higher for FDE compared with CON, but FDEPRO, PRO was not different to FDEPRO and CON (*P* < 0.001). At 72 h, FDEPRO, FDE was not different from each other but higher than CON; however, PRO was not different from all other treatments (*P* < 0.001).

**Table 5.** Effects of FDE with or without protease on AD of DM, NDF, and ADF at different timepoints of steeped WM

Item (%)		DM	NDF	ADF
Treatment^1^*Time^2^				
CON	0	1.45^g^	13.07^e^	13.99^e^
CON	12	7.25^ef^	13.15^e^	15.20^de^
CON	24	7.05^f^	14.38^e^	12.91^e^
CON	48	8.75^def^	19.17^de^	14.46^de^
CON	72	7.36^ef^	17.79^e^	17.29^cde^
FDE	0	1.88^g^	42.21^a^	17.06^cde^
FDE	12	7.97^def^	43.58^a^	17.82^bcde^
FDE	24	9.64^bcd^	44.09^a^	28.12^ab^
FDE	48	11.50^ab^	40.35^a^	26.94^abc^
FDE	72	11.86^a^	42.74^a^	28.98^a^
PRO	0	1.62^g^	25.99^cd^	13.28^e^
PRO	12	8.95^cdef^	27.13^cd^	17.99^bcde^
PRO	24	10.94^abc^	27.45^c^	15.58^de^
PRO	48	11.22^ab^	29.05^bc^	16.73^cde^
PRO	72	11.34^ab^	35.94^ab^	22.44^abcde^
FDEPRO	0	1.57^g^	43.13^a^	15.95^de^
FDEPRO	12	9.39^bcde^	42.35^a^	21.97^abcde^
FDEPRO	24	11.49^ab^	40.83^a^	26.84^abc^
FDEPRO	48	12.04^a^	39.57^a^	24.60^abcd^
FDEPRO	72	13.04^a^	43.29^a^	29.50^a^
SEM		0.53	2.33	3.18
Treatment				
CON		6.37	15.51	14.77
FDE		8.57	42.59	23.78
PRO		8.81	29.11	17.21
FDEPRO		9.52	41.83	23.77
SEM		0.37	1.84	2.60
Time, h				
0		1.63	31.10	15.07
12		8.39	31.55	18.24
24		9.78	31.69	20.86
48		10.88	32.04	20.68
72		10.90	34.94	24.55
SEM		0.38	1.87	2.64
*P*-value				
Treatment		<.0001	0.0067	0.0224
Time		<.0001	<.0001	<.0001
Treatment*Time		<.0001	0.0074	<.0001

^1^CON; no enzyme, PRO; CON + protease, FDE; CON + FDE, and FDEPRO: CON + FDE and protease.

Enzyme activities were: FDE: xylanase: 62,000 U/g, β-glucanase: 8,000 U/g, cellulase: 37,000 U/g, proteases: acid 2,500,000 U/g, neutral 800,000 U/g, and alkaline 1,300,000 U/g.

About 50 g of DDGS or WM was mixed with 500 mL of deionized water and 0.5 g of respective enzyme and incubated at 37 °C with agitation.

^2^0; steeped for 0 h followed by 12 h of settling at 4 °C,12; steeped for 12 h followed by 12 h of settling at 4 °C, 24;steeped for h followed by 12 h of settling at 4 °C, 48; steeped for 48 h followed by 12 h of settling at 4 °C, 72; steeped for 0 h followed by 72 h of settling at 4 °C. At each timepoint, LSmeans with different letters differs (*P* < 0.05), *n* = 8.

## DISCUSSION

The chemical composition of DDGS and WM used in the present study was comparable to the values presented in other publications (Salim et al., 2010; Rosenfelder et al., 2013; Huang et al., 2017; Casas et al., 2018). As NDF fraction includes hemicellulose, cellulose, lignin, and ADF cellulose and lignin, hemicellulose can be calculated. Hemicellulose is a heteropolysaccharide complexed with cellulose fibrils in plant cell walls (Cui et al., 2011) and is more easily hydrolyzed to constituent monosaccharides than cellulose (Wyman et al., 2005). Arabinoxylans, arabinogalactans, xyloglucans, xylans, and mixed-linkage glucans are main hemicelluloses in cereal grains (de Lange, 2000; Cui et al., 2011). Hemicellulose concentration was 2.27 times greater for DDGS than in corn and 2.04 times greater for WM than in wheat. This can be explained by co-product production processes. Ethanol is produced by fermenting starches in grains; therefore, DDGS typically contains higher concentrations of nonfermentable constituents such as fat and protein (Woyengo et al., 2016). The fiber concentration in WM is also greater than in wheat because it is a mixture of wheat bran, shorts, germ, flour, and some of the “tail of the mill” residues (Erickson et al., 1985). Therefore, co-products from wheat flour milling contain a higher concentration of fiber than parent grain (Widyaratne and Zijlstra, 2007). The monosaccharides concentration in DDGS showed greater xylose, arabinose, mannose, and galactose, and in WM, greater xylose, arabinose and galactose contents were seen relative to grains. However, grains had a greater concentration of glucose which may be reflecting the higher starch content. The glucose concentration in WM was lower than in wheat; however, a considerable amount of glucose was still found in WM. This may be because WM still has high starch content due to “contamination” of mixed fractions with endosperm tissue as reflected in the high glucose content of this fraction.

In most cereal-based ingredients, the concentration of non-starch polysaccharide (**NSP**) is high and is especially rich in arabinoxylans, cellulose, β-glucans, xyloglucans, xylans, and arabinogalactans (Bach Knudsen, 1997; de Lange, 2000; Rumpagaporn et al., 2015). Arabinoxylans are hemicelluloses consisting of β-1,4-linked d-xylose residues substituted with arabinose side chains (Lu et al., 2000) and are abundantly found in the cell walls of corn and wheat (Bach Knudsen, 1997). Therefore, the higher xylose and arabinose concentration relates to the higher concentration of arabinoxylan in co-products than in the grains. The degree of substitution of arabinose depends on the cereal species and cell wall types, which affect the solubility of the arabinoxylan and the susceptibility to enzymatic hydrolysis (Bach Knudsen, 1997, 2014). The A/X can give an idea of the degree of arabinose substitution. Therefore, the arabinose to xylose ratio (A/X) is an important parameter. The A/X of DDGS and WM was 0.73 and 0.62 from a study conducted by Jaworski et al. (2015), which was lower than the value presented in the current study. The difference can be due to inherent quantitative differences in the concentration of arabinose and xylose due to the variety and growing condition of the parent grains (Pedersen et al., 2014). In general, cereal bran has a higher A/X than other parts of grains; however, corn bran has a greater A/X compared to wheat bran (Zhang et al., 2014). The A/X was higher for DDGS and WM than in corn and wheat pointing to a more densely substituted structure in DDGS and WM (de Vries et al., 2013; Pedersen et al., 2014). These more densely substituted structures are harder to hydrolyze and ferment.

More 5-Ara-f linkages in co-products suggested that more ferulic acid residues were attached to arabinoxylans. Ferulic acids are indeed mainly linked to the fifth carbon of the arabinose side chain of arabinoxylan (Buanafina, 2009). Highly feruloylated arabinoxylans are more resistant to hydrolytic degradation and fermentation (Snelders et al., 2014). This is caused by cross-linking through diferulic acid bridges of the polysaccharide chains and the associated formation of lignin–ferulate–xylan complexes (Buanafina, 2009), which decrease the solubility. The 3,4-Glc, 4,6-Glc, and 3,4,6-Glc linkage contents were higher in grains than co-products indicating richer starch content. The 4-xylose (**Xyl**) linkage concentration was higher in WM, whereas 3,4-Xyl linkages were only detected in DDGS. This suggested that corn, wheat, and WM have more linear 1,4-linked xylose structures compared with DDGS. Branched structures can be estimated by analyzing terminal glycosyl linkages. Greater T-Ara-f content in DDGS is another indication of a more densely substituted structure of arabinoxylan as compared with the arabinoxylan molecules found in the other ingredients. The T-Gal linkage stems from arabinogalactans that are present in cereals but the higher concentration in co-products. Arabinogalactan is a proteoglycan formed by a backbone of 1–4 linked galactose residues carrying arabinose substitutions at the O-3 positions of a select part of the backbone galactose residues (Cipriani et al., 2009; Bader Ul Ain et al., 2018). Studies have reported that a considerable amount of arabinogalactans are found in cereal hulls and husks (Saeed et al., 2011); therefore, the increased levels of T-Gal in co-products compared with corn and wheat was expected.

The efficacy of the FDE is dependent on types of grain and ingredients (Myers and Patience, 2014; Kiarie et al., 2016b). In the present study, FDE and FDEPRO treatments increased arabinose concentration at 12 h in DDGS and xylose in WM. The majority of fiber fractions in DDGS are arabinoxylans that are cross-linked with arabinofuranosyl or glucuronic acid residues which can potentially prevent xylanase from accessing xylan backbones (de Vries et al., 2013). As shown in the present study, the A/X ratio and glycosyl linkage of arabinose of DDGS and WM was 1.33, 2.52, 2.30, and 1.02, 0.68, 1.36, respectively, which suggested a more complex structure in DDGS (Pedersen et al., 2014). de Vries et al. (2013) reported that when DDGS was treated with a combination of enzymes and different processing techniques, such as wet milling, extrusion, autoclaving, and hydrothermal acid treatment, the cell walls were barely affected; therefore, FDE may have been less effective in DDGS. Glucose concentration for CON, PRO, FDE, and FDEPRO at 12 h was 5.36, 6.34, 37.19, and 31.16 for DDGS and 5.54, 41.26, 38.73, and 56.85 for WM, respectively. Greater glucose concentration compared with arabinose concentrations seen in FDE treatments may be because β-glucans and to some extent cellulose are easily hydrolyzed to compared with arabinoxylans (Karppinen et al., 2000).

At 0 h, the concentration of monosaccharides in the supernatant were greater in steeped WM than in supernatant of steeped DDGS, which may be due to wheat arabinoxylan being more soluble or because WM does not undergo an ethanol extraction process where carbohydrates are extracted. Izydorczyk and Biliaderis (1995) reported that wheat arabinoxylans are more soluble than arabinoxylans found in barley, oat, and corn. The concentration of arabinose, xylose, and glucose was similar between CON and PRO in steeped DDGS; however, in steeped WM, PRO had greater xylose and glucose concentrations than CON. This can be explained by the complex protein–fiber structure of DDGS perhaps linked to heating processes. Heat treatment during ethanol production forms protein–fiber complexes due to Maillard reactions (Jha et al., 2015). The increase of monosaccharide concentrations at 12–24 h followed by a decrease at 48 to 72 h can be explained by microbes utilizing these monosaccharides for energy source while producing organic acids. It has been well reported that natural fermentation of cereal grains results in carbohydrates and other indigestible poly and oligosaccharide concentrations to decrease (Blandino et al., 2003).

The hydrolysis, biochemical metabolism, and microbial activity during cereal fermentation results in organic acids as end products (Blandino et al., 2003; Rho et al., 2018b, 2019). Heterofermentative organisms are known to be the predominant endogenous microbial flora in grains (Nanson and Fields, 1984; Khetarpaul and Chauhan, 1989). The different trends of organic acid concentrations over time in steeped DDGS vs. steeped WM may be related to varying types and complexity of glycosyl linkages, monosaccharide arrangement and types, and solubility and physical properties of dietary fibers as these factors can affect the fermentation rate (Wang et al., 2019). In the present study, greater glucose concentration in WM than in DDGS may have influenced the higher lactic acid concentration in steeped WM. However, other factors such as different microbes and enzymes inherent to the material can also cause different outcomes (Canibe and Jensen, 2012). Regardless of enzymes of application and different co-products, the decrease in pH and increase of lactic and acetic acid concentrations over time as seen in the current study agrees with past fermentation/incubation studies (Mikkelsen and Jensen, 2000; Dujardin et al., 2014; Wiseman et al., 2017; Rho et al., 2018a,b). Mikkelsen and Jensen (2000) observed that the pH was reduced below 4.5 in both liquid feeds that were fermented with or without microbial inoculums. This is because as soon as feedstuff is soaked with water, naturally occurring enzymes, bacteria and yeasts start to produce lactic and acetic acid (which reduce the pH), and ethanol (Canibe and Jensen, 2012). Therefore, reduced pH and increased lactic and acetic acid in all the treatments over time was expected.

The AD of DM for CON, PRO, FDE, and FDEPRO at 72 h for DDGS was 9.49, 9.26, 11.04, and 11.58 and for WM, 7.36, 11.34, 11.86, and 13.04, respectively. Greater AD of DM for FDEPRO compared with FDE in both co-products may be due to increased protein degradation (Pedersen et al., 2015). However, this could not be ascertained in the present study as the disappearance of CP was not determined. In DDGS, the AD of NDF and ADF for CON, PRO, FDE, and FDEPRO at 72 h was 3.43, 5.40, 20.36, 19.39 and 9.8, 5.02, 27.6, 28.57, respectively. In WM the AD of NDF and ADF for CON, PRO, FDE, and FDEPRO at 72 h was 17.79, 35.94, 42.74, and 43.29 and 17.29, 22.44, 28.98, and 29.50. Compare to CON, this reflected more disappearance of hemicellulose such as arabinoxylans, in DDGS than in WM. However, as mentioned previously, wheat arabinoxylans are more soluble than arabinoxylans found corn (Izydorczyk and Biliaderis, 1995); therefore, in steeped WM, the difference in AD of NDF and ADF between CON and enzyme-treated samples may not have been as great as in steeped DDGS, as soaking WM in water has been shown to lead to a considerable breakdown of fiber (Moran et al., 2016). Xylanase inhibitors that are more concentrated in wheat than other cereals can reduce the xylanase activity (Elliott et al., 2003; Gusakov, 2010). However, in the current study, the xylanase inhibitor levels in co-products were not measured. Nonetheless, the AD of NDF and ADF along with monosaccharides release indicated FDE was effective in hydrolyzing wheat fiber; therefore, we can predict that the xylanase inhibitors were not an issue when FDE was included in steeped WM.

Applying FDE mixture increased arabinose, xylose, and glucose release concomitant with the disappearance of NDF and ADF fractions in both DDGS and WM at certain timepoints. The FDE mixture used in the present study contained xylanase, β-glucanase, and cellulase activities to target arabinoxylan, β-glucan, and cellulose components in cereal co-products. However, different trends seen in organic acids and monosaccharide concentrations in DDGS and WM may be due to differences in fiber structure as we can be related to concentrations of monosaccharides and glycosyl linkages. Therefore, when applying the same enzymes to different co-products, the magnitude of response can vary, and the enzyme mix should be aligned with the specific feed composition, and steeping hour should be carefully chosen. However, steeping DDGS and WM with FDE mixture containing xylanase, β-glucanase, and cellulase activities were effective in hydrolyzing fiber components and release monosaccharides in cereal co-products at 12, 24, or 72 h of steeping. In general, protease, alone or in combination with FDE, had only limited effects on fiber hydrolysis, suggesting a minor role of protease in increasing hydrolysis on fiber under conditions, despite steeping time in the present study. Further research is warranted to validate the in vivo effects of FDE, particularly in solubilizing fiber fractions in co-products that are typically resistant to gastrointestinal microbial fermentation.
